# Heavy Drinking in College Students Is Associated with Accelerated Gray Matter Volumetric Decline over a 2 Year Period

**DOI:** 10.3389/fnbeh.2017.00176

**Published:** 2017-09-29

**Authors:** Shashwath A. Meda, Alecia D. Dager, Keith A. Hawkins, Howard Tennen, Sarah Raskin, Rebecca M. Wood, Carol S. Austad, Carolyn R. Fallahi, Godfrey D. Pearlson

**Affiliations:** ^1^Olin Neuropsychiatry Research Center, Hartford HealthCare Corporation, Hartford, CT, United States; ^2^Department of Psychiatry, Yale University, New Haven, CT, United States; ^3^Department of Community Medicine, University of Connecticut School of Medicine, Farmington, CT, United States; ^4^Department of Psychology and Neurosciences, Trinity College, Hartford, CT, United States; ^5^Department of Psychology, Central Connecticut State University, New Britain, CT, United States; ^6^Department of Neuroscience, Yale University, New Haven, CT, United States

**Keywords:** substance use, college, binge, morphometry, cortical

## Abstract

**Background:** Heavy and/or harmful alcohol use while in college is a perennial and significant public health issue. Despite the plethora of cross-sectional research suggesting deleterious effects of alcohol on the brain, there is a lack of literature investigating the longitudinal effects of alcohol consumption on the adolescent brain. We aim to probe the longitudinal effects of college drinking on gray matter change in students during this crucial neurodevelopmental period.

**Methods:** Data were derived from the longitudinal Brain and Alcohol Research in College Students (BARCS) study of whom a subset underwent brain MRI scans at two time points 24 months apart. Students were young adults with a mean age at baseline of about 18.5 years. Based on drinking metrics assessed at both baseline and followup, subjects were classified as sustained abstainers/light drinkers (*N* = 45) or sustained heavy drinkers (*N* = 84) based on criteria established in prior literature. Gray matter volumetric change (GMV-c) maps were derived using the longitudinal DARTEL pipeline as implemented in SPM12. GMV-c maps were then subjected to a 1-sample and 2-sample *t*-test in SPM12 to determine within- and between-group GMV-c differences in drinking groups. Supplementary between-group differences were also computed at baseline only.

**Results:** Within-group analysis revealed significant decline in GMV in both groups across the 2 year followup period. However, tissue loss in the sustained heavy drinking group was more significant, larger per region, and more widespread across regions compared to abstainers/light drinkers. Between-group analysis confirmed the above and showed a greater rate of GMV-c in the heavy drinking group in several brain regions encompassing inferior/medial frontal gyrus, parahippocampus, and anterior cingulate. Supplementary analyses suggest that some of the frontal differences existed at baseline and progressively worsened.

**Conclusion:** Sustained heavy drinking while in college was associated with accelerated GMV decline in brain regions involved with executive functioning, emotional regulation, and memory, which are critical to everyday life functioning. Areas of significant GMV decreases also overlapped largely with brain reward and stress systems implicated in addictive behavior.

## Introduction

Alcohol is the most widely used intoxicant among adolescents and young adults, with rates of binge drinking and alcohol use disorders peaking between ages 18 and 25 (SAMHSA, 2016), a period commonly referred to as emerging adulthood (Arnett, 2000). Recent national surveys revealed that 39% of 18–25-year-olds reported past-month binge drinking, compared to 25% of adults ages 26 and older (SAMHSA, 2016). This age range also encompasses the final stages of neuromaturation (Gogtay et al., 2004), which may be negatively affected by heavy alcohol use.

Age-related refinements in brain structure and function continue well into emerging adulthood. Region-specific patterns of cortical thinning and gray matter volume reduction have been identified, with higher order association cortices, including prefrontal and temporal regions, maturing last, and varying by sex (Gogtay et al., 2004). Measureable changes in gray matter volume have been observed over a 6-month period in first-year college students, between fall and spring semesters (Bennett and Baird, 2006). These neuromaturational changes are thought to underlie cognitive improvements throughout this age range. In particular, executive functions, subserved by late-developing frontal lobe regions, show dramatic enhancements in late adolescence and emerging adulthood (Casey et al., 2000).

Several neuroimaging investigations have characterized structural brain differences among adolescent drinkers, yet fewer investigations have focused on emerging adults. Extant research has most consistently implicated frontal lobe abnormalities among adolescent drinkers (Silveri et al., 2016). Cross sectional studies have revealed smaller volumes and thinner cortices among adolescent heavy drinkers, particularly in frontal, temporal, and cingulate regions (De Bellis et al., 2005; Medina et al., 2008; Fein et al., 2013; Whelan et al., 2014), with greater alcohol consumption associated with larger volume reductions (De Bellis et al., 2005). A recent voxel-based morphometry study of young adults ages 22–28 identified less gray matter volume among heavy drinkers in anterior cingulate, orbitofrontal, temporal, and insula cortices (Heikkinen et al., 2017). Longitudinal investigations have examined adolescents both before and after the onset of heavy drinking, and described steeper declines in cortical thickness in prefrontal (Luciana et al., 2013; Squeglia et al., 2015) and temporal (Squeglia et al., 2014, 2015) regions in alcohol initiators. One diffusion tensor imaging study ascertained white matter integrity in binge drinkers and controls during late adolescence, and again 3 years later during emerging adulthood. Lower fractional anisotropy was observed at baseline among binge drinkers throughout widespread brain regions (Jacobus et al., 2009; McQueeny et al., 2009), many of which showed additional decline among those who continued binge drinking during the 3-year follow-up period (Jacobus et al., 2013). However, to our knowledge, this is the first longitudinal study to examine trajectories of gray matter volume development in an attempt to shed more light on implications of continued heavy drinking among emerging adult drinkers.

To delineate the influence of ongoing heavy alcohol use on brain gray matter development in emerging adulthood, we collected structural magnetic resonance imaging (MRI) scans at two time-points in a college cohort, at approximately age 18 and again ~2 years later at age 20. We conducted longitudinal voxel based morphometry (VBM) analyses in individuals who were either abstainers/light drinking controls or heavy drinkers at both time-points (i.e., sustained light use or sustained heavy use). Given that cortical pruning is part of the normal developmental process at this age, we predicted that both groups would show significantly reduced gray matter over time; however, sustained heavy drinkers would show a greater decline in gray matter volume compared to sustained light drinkers, especially in frontal and temporal brain regions.

## Materials and methods

### Participants

A cohort of first-year students (age range 18–23; mean 18.5 years) was recruited from two local colleges with diverse populations through e-mail, flyers and classroom visits to solicit participation in the Brain and Alcohol Research in College Students (BARCS) study (Ahmadi et al., 2013; Dager et al., 2014; Worhunsky et al., 2016; Meda et al., 2017). The recruitment captured greater than 95% of possible participants. All subjects provided written informed consent to participate in the study. The study was conducted in accordance with the declaration of Helsinki and was approved by institutional review boards at Central Connecticut State University (CCSU), University of Connecticut, Trinity College, Hartford Hospital, and Yale University.

A representative sub-sample of 200 individuals, all free from MRI contraindications, underwent a neuroimaging battery including structural imaging scans at baseline and follow up (24 months apart on average). Participants were divided into two groups at both baseline and 24-month follow-up based on drinking quantity and frequency (also see below section), similar to definitions used in previous studies (Dager et al., 2014; Worhunsky et al., 2016). Heavy drinking was defined as binge drinking (≥4 drinks/occasion for females, ≥5 drinks/occasion for males) ≥13 of the past 26 weeks, or meeting criteria for alcohol abuse. Light drinking included teetotalers or binge drinking <13 of the previous 26 weeks and never meeting criteria for alcohol abuse. The final sample included 139 participants: (i) 55 light drinkers who either abstained or sparingly drank at baseline and follow-up and (ii) 84 heavy drinkers, who drank heavily at baseline and follow-up (Please see Table 1). The rest of the participants were excluded from the study due to one of the following reasons (a) not scanned at one of the two time points, (b) scans did not pass quality control at one or both time points (for e.g., excessive motion, bad scan quality, other artifacts etc.) and (c) could not be categorized into one of the above drinking categories at one or both time points and (d) had valid scans at both time points but were neither sustained abstainer/light or heavy drinkers.

**Table 1:** Demographics and clinical characteristics of drinking groups investigated in the study.

**Subject demographics**	**Sustained light users**		**Sustained heavy users**		**Statistic**	
	**(Total *N* = 55)**		**(Total *N* = 84)**			
	**Mean**	***SD***	**Mean**	***SD***	***t***	***p*-value**
Age at Baseline (years)	18.38	0.59	18.48	0.81	0.16	NS
Baseline State-Trait Anxiety Inventory (STAI)	42.46 (*N* = 41)	10.12	54.42 (*N* = 49)	12.32	−0.78	NS
Baseline Beck Depression Inventory (BDI)	4.12 (*N* = 41)	6.27	5.22 (*N* = 5 1)	5.56	−0.88	NS
Number of Monthly Drinks (Baseline)	2	5	48	48	−7.18	<0.001
Number of Monthly Drinks (Follow up)	3	5	40	42	−6.54	<0.001
Time Elapsed Between Scans (Months)	24.39	5.02	25.2	5.23	−0.90	NS
Baseline Clinical Characteristics	***N***		***N***		**Chi-square**	***p*****-value**
**GENDER**						
Male	22		49		4.47	0.04
Female	33		35			
**CIGARETTE SMOKER**						
No	38		49		1.51	NS
Yes	3		1			
Missing	14		34			
**FAMILY HISTORY FOR ALCOHOLISM**						
Negative	28		34		0.03	NS
Positive	13		17			
Missing	14		33			
**ADHD**						
No	36		47		3.74	NS
Yes	3		0			
Missing	16		37			
**MDD (MINI)**						
No	54		78		1.97	NS
Yes	1		6			
**AGORAPHOBIA (MINI)**						
No	54		78		1.97	NS
Yes	1		6			
**PANIC DISORDER (MINI)**						
No	40		48		0.65	NS
Yes	1		3			
Missing	14		33			

Exclusion criteria included current schizophrenia or bipolar disorder, history of seizures or significant head injury, not meeting our criteria for drinking groups (described in the assessments section below), and excessive motion (visual evaluation by imaging expert SAM) during scanning.

### Assessments

At both baseline and follow-up, past 6-month alcohol use and abuse diagnosis was assessed using an in-house interview incorporating items from the Semi-Structured Assessment for the Genetics of Alcoholism (SSAGA; Bucholz et al., 1994) and the alcohol use module from the Structured Clinical Interview for the DSM-IV (SCID; First et al., 1994). In addition at baseline, we collected a variety of variables related to psychiatric status and family history of alcoholism (FHA). Students were administered a select section of the Barkley Adult ADHD Rating Scale–IV (BAARS-IV) to assess ADHD symptoms (Barkley et al., 2011). Students were asked to self-report whether they had ever been formally diagnosed with ADHD and whether they were currently receiving treatment for the same. We coded individuals as having a history of ADHD if they responded “yes” to both questions. All participants also received the M.I.N.I. structured psychiatric diagnostic assessment (Sheehan et al., 1998) (https://medical-outcomes.com/). We found three prevalent diagnoses including major depressive disorder (MDD), agoraphobia and panic disorder among our college sample, which were binary coded (yes/no) and used for further analyses. FHA was assessed using the Family History Assessment Module (FHAM) (Rice et al., 1995). Students completed the trait sections of the State Trait Anxiety Index (STAI) questionnaire (Spielberger et al., 1983). A total STAI sum score was calculated, normed based on gender and was used for further analysis. Students were also administered the Beck Depression Inventory (BDI) to assess depressive symptoms at study entry (Beck et al., 1961); all items from the inventory were summed to yield a total score. At each scan time point, participants provided urine toxicology samples for negative screening of commonly abused substances, negative alcohol breathalyzer screens, and negative urine pregnancy screens (females).

### Image acquisition and processing

Magnetic resonance structural brain images were collected on a Siemens Allegra 3T system (Siemens AG, Erlangen, Germany) located at the Olin Neuropsychiatric Research Center in Hartford, CT. Images were collected using a sagittal T1 MPRAGE sequence with the following parameters TR/TE/TI = 2,300/2.74/900 ms, flip angle = 8°, slab thickness = 176 mm, FOV = 176 × 256 mm, matrix = 176 × 256 × 176, voxel size = 1 mm^3^, pixel band-width = 190 Hz, scan time = 10:09.

### Computation of longitudinal gray matter volume (GMV) change

In order to compute gray matter volume change maps, structural images from each time point were subjected to a technique called symmetric diffeomorphic modeling of longitudinal data, implemented in SPM12 (Ashburner and Ridgway, 2012). The following steps were implemented to derive the volumetric rate of change maps for each subject (1) a mid-point image was estimated by optimally mapping the template with each time point image by means of a groupwise-consistent 3D non-linear image registration technique. A Jacobian difference image was then estimated that records the difference for deformation from the mid-point image to the first scan and this to the second scan. This Jacobian difference map was further divided by the time elapsed between the two scans to derive a rate of change map. (2) the mid-point image was then segmented into their respective GM, WM, and CSF constituents. (3) GM images from step (2) were used to create a study-specific template using DARTEL. (4) The Jacobian rate of difference images from step (1) were multiplied by the GM image from step (2) to derive a GMV rate of change map (GMV-c). (5) The images from step (4) were normalized to MNI space and smoothed with a 6 mm FWHM Gaussian kernel. (6) Images derived from step (5) were then subjected to further statistical analyses as described below.

### Statistical analyses

Continuous demographic variables were compared between groups using an independent sample *t*-test in SPSS v21 (https://www.ibm.com/analytics/us/en/technology/spss/). Categorical variables were examined with a chi-square analysis using the same software.

The GMV-c maps were subjected to a 1-sample *t*-test in SPM12 (separately for light and heavy drinkers) to detect significant within-group decreases or expansions over the 2-year follow-up period on a voxel-by-voxel basis across the whole brain. Further, we carried out a 2-sample *t*-test (adjusted for sex) within the general linear model framework to assess GMV-c differences across light and heavy drinking groups (group by time interaction). Given that alcohol may influence brain changes differentially by sex (Squeglia et al., 2014) supplementary analyses were conducted to look for any possible group-by-sex or group-by-sex-by-time interactions. In addition to the above, to assess if groups differed in GMV at baseline, we conducted a follow-up 2-sample *t*-test adjusted for sex and total intracranial volume using only GMV calculated at the first time point. Although the major aim of the paper was to capture and report longitudinal changes, this explicit baseline analysis provides readers a better understanding of what volumetric changes already existed at baseline and how these brain structures change over time. All the above statistical analyses were conducted using permutation based analyses coupled with corrections for whole brain multiple comparisons using the threshold free cluster enhancement technique (TFCE) as implemented in the TFCE toolbox (http://dbm.neuro.uni-jena.de/tfce/). Resulting maps were thresholded at the *p* < 0.05 family wise error (FWE) whole brain corrected level and displayed using the NeuroElf toolbox (http://neuroelf.net/).

## Results

Groups did not differ by age, smoking, FHA or any MINI diagnostic status. Groups also had similar scores on the STAI and BDI. However, groups differed by sex (chi-square = 4.47; *p* = 0.03). Complete description of demographics and clinical characteristics of study participants are provided in Table 1.

**Figure 1:**
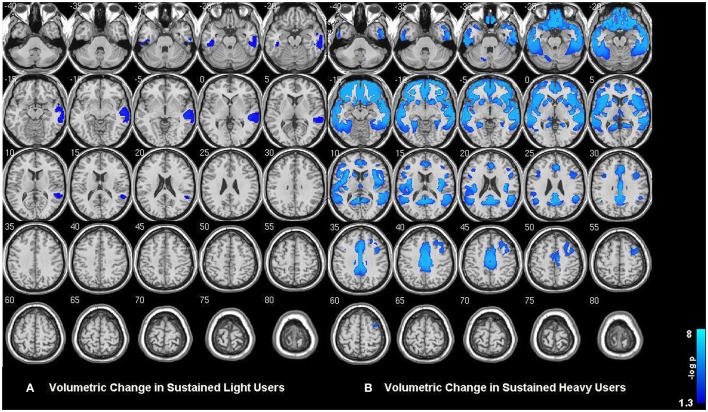
**(A)** Gray matter volumetric change in sustained abstainers/light and **(B)** sustained heavy users across the two year period. Results are displayed at *p* < 0.05 FWE corrected across the whole brain. Images are on a negative log *P* scale. Note that both groups only showed significant decline and no expansions.

**Figure 2:**
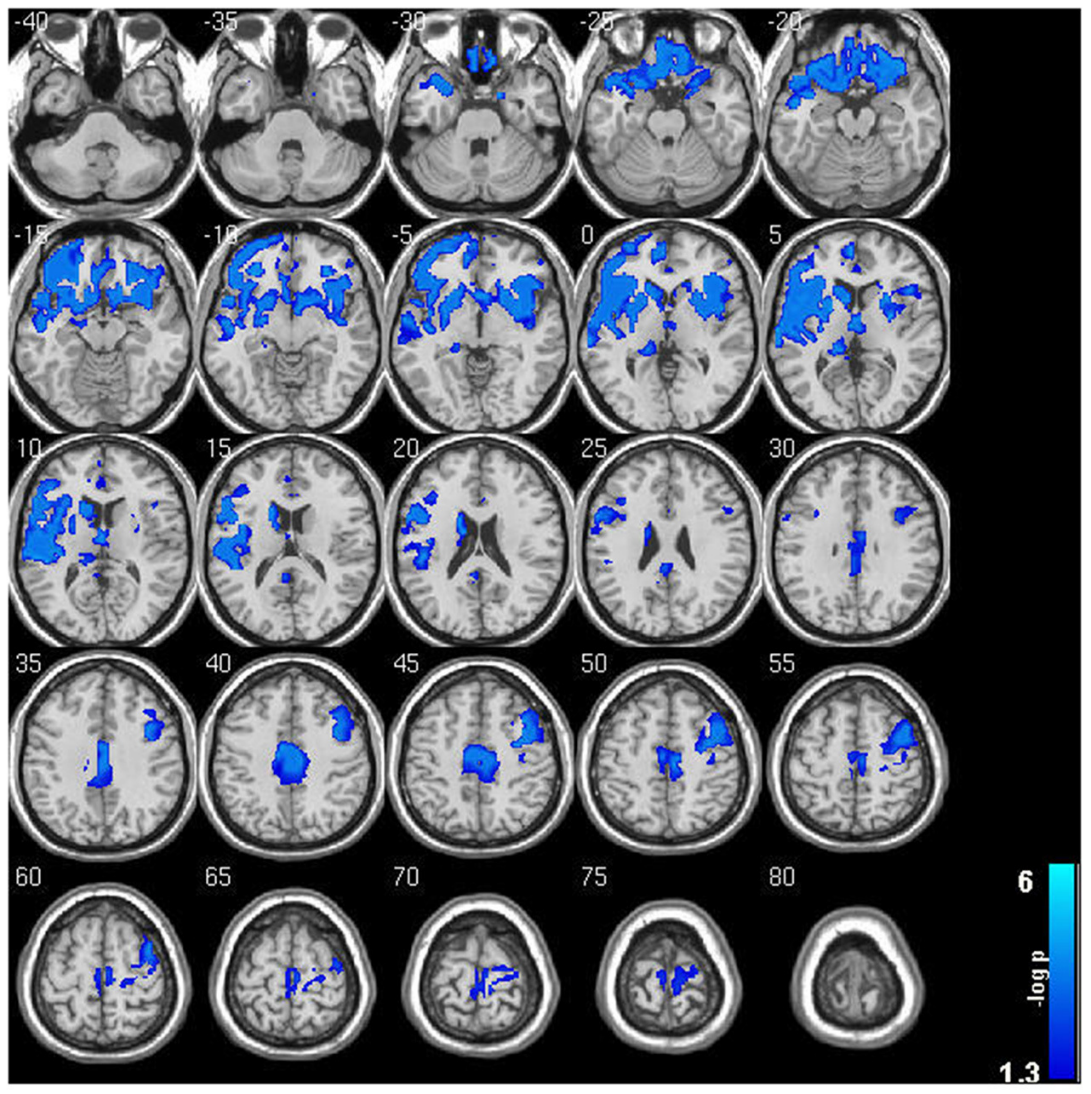
Significant gray matter volumetric change between sustained abstainers/light and sustained heavy user groups across the 2 year period. Note the widespread accelerated GMV decreases in sustained heavy users of alcohol compared to their light user peers. Results are displayed at *p* < 0.05 FWE corrected across the whole brain. Images are on a negative log *P* scale.

**Figure 3:**
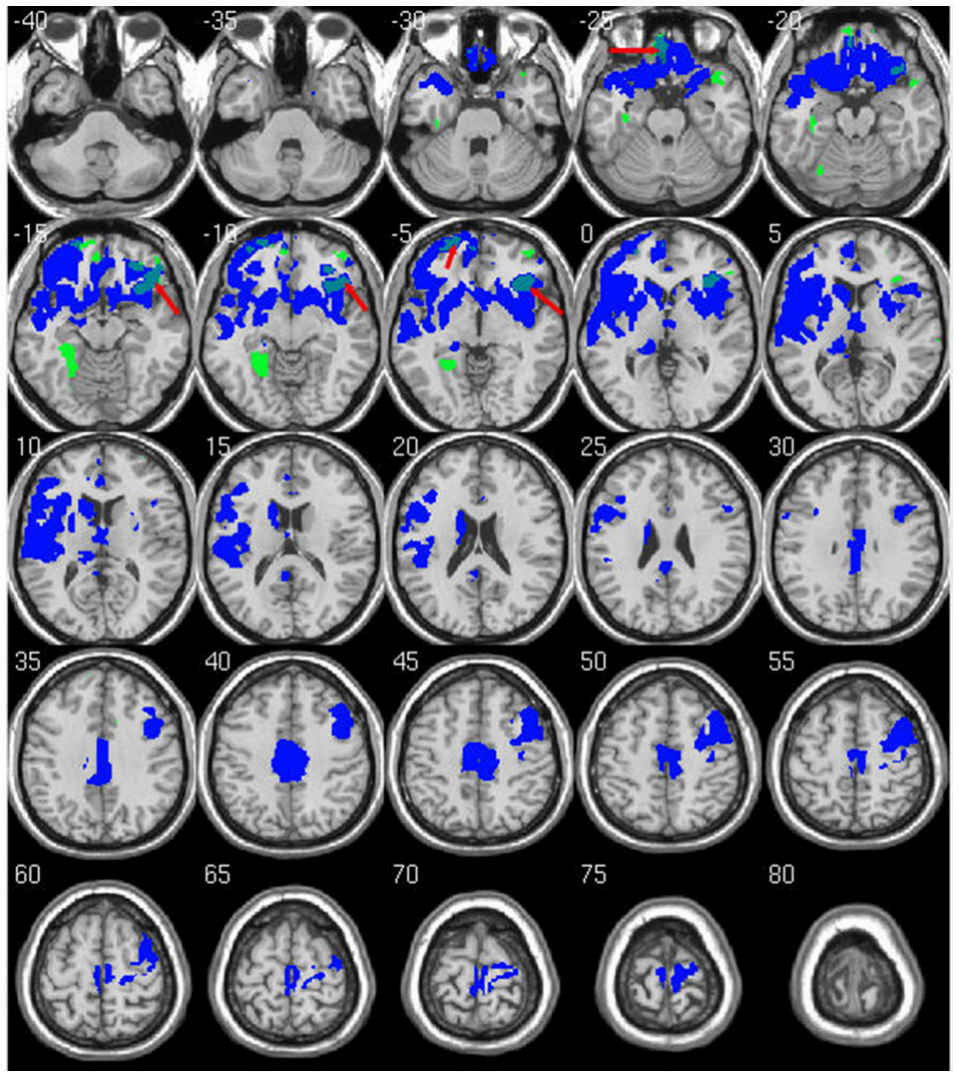
Overlap of significant between-group differences at baseline and longitudinal follow up. Regions marked in green show lower GMV in heavy drinking group compared to abstainers/light at baseline. Regions in blue are the same longitudinal results as shown in Figure 2. Overlap between baseline and longitudinal differences are shown in light blue (red arrows). Results are displayed at *p* < 0.05 FWE corrected across the whole brain. Images are on a negative log *P* scale.

Within-group analysis revealed significant GMV loss in both drinking groups over time (see Figure 1). No significant regional expansion of GMV over time was detected in either group. GMV decline in stable light users was mostly localized to inferior/middle temporal and fusiform gyrus. Gray matter decreases in individuals with sustained heavy alcohol use were lager in magnitude, and included the above regions plus several other brain regions including inferior/middle frontal gyrus (I/MFG), anterior cingulate, insula, thalamus, caudate, and parahippocampal gyri. Longitudinal GMV differences assessed across groups (see Figure 2) showed the heavy use group to have a significantly increased rate of gray matter decline, primarily in fronto-striatal regions, including inferior/medial frontal, anterior cingulate, parahippocampus, precentral gyrus, and insula. A complete description of within-group and between-group results is presented in Tables 2A,B. Further, we detected no group-by-sex or group-by-sex-by-time interactions at the whole brain level. Exploratory analysis of between-group baseline differences in GMV revealed significantly decreased GMV in the heavy drinking group in a subset of regions that also showed exaggerated decline across time. Overlap of regions was mainly localized to superior, medial and inferior frontal gyri after correction for multiple comparisons. Regions showing baseline differences are visually represented in Figure 3.

**Table 2:** Significant regions of GMV decreases for both **(A)** within- and **(B)** between-group analyses.

**(Hemisphere) Region**	**Brodmann area**	***x***	***y***	***z***	**Max (−log p)**	**Cluster size (mm^3^)**
**(A) WITHIN-GROUP RESULTS**						
**Sustained light users**						
LH Fusiform Gyrus	20	−45	−30	−24	1.71	170
LH Inferior Temporal Gyrus	20	−60	−27	−30	1.33	10
RH Fusiform Gyrus	20	51	−27	−24	1.76	61
RH Inferior Temporal Gyrus	20	57	−30	−15	1.79	165
RH Middle Temporal Gyrus	21	54	−21	−6	2.13	827
**Sustained heavy users**						
LH Anterior Cingulate	32	0	39	6	3.70	76.41
LH Caudate	Caudate body	−12	15	9	3.10	45.36
LH Claustrum		−27	24	9	3.70	148.23
LH Fusiform Gyrus	20	−54	−21	−21	3.70	152.82
LH Fusiform Gyrus	37	−39	−45	−15	3.22	118.26
LH Inferior Frontal Gyrus	11	−18	36	−18	3.70	153.09
LH Insula	13	−36	0	15	3.70	99.36
LH Medial Frontal Gyrus	10	−3	54	0	3.70	116.91
LH Middle Frontal Gyrus	46	−36	27	21	1.84	14.58
LH Middle Frontal Gyrus	9	−33	12	30	1.57	11.88
LH Middle Occipital Gyrus	19	−45	−60	−6	2.18	63.45
LH Middle Temporal Gyrus	21	−45	−33	0	3.70	93.96
LH Middle Temporal Gyrus	37	−42	−63	6	1.71	14.85
LH Pyramis	−	−15	−63	−27	1.31	13.5
LH Superior Temporal Gyrus	38	−48	12	−9	3.70	68.04
LH Superior Temporal Gyrus	22	−48	−15	−9	3.70	69.39
LH Superior Temporal Gyrus	39	−42	−51	21	3.22	79.11
LH Superior Temporal Gyrus	41	−54	−30	9	2.49	44.82
LH Thalamus	−	−12	−33	3	1.79	12.69
LH Thalamus	Medial dorsal nucleus	0	−15	9	1.76	13.23
RH Anterior Cingulate	32	12	48	−9	3.70	118.8
RH Anterior Cingulate	25	3	18	−9	3.70	120.69
RH Cingulate Gyrus	24	12	−15	42	3.70	118.8
RH Cingulate Gyrus	31	6	−45	27	3.70	109.89
RH Cingulate Gyrus	32	3	21	36	3.70	57.78
RH Fusiform Gyrus	37	36	−39	−12	2.49	66.96
RH Inferior Frontal Gyrus	47	48	24	−3	3.70	199.8
RH Inferior Frontal Gyrus	45	42	18	9	3.70	88.56
RH Inferior Frontal Gyrus	44	51	12	18	3.10	27
RH Insula	13	42	−18	3	3.70	44.01
RH Medial Frontal Gyrus	25	9	6	−15	3.40	26.19
RH Medial Frontal Gyrus	9	3	33	30	3.40	44.01
RH Middle Frontal Gyrus	10	42	57	−9	3.70	129.87
RH Middle Frontal Gyrus	47	51	42	−6	3.40	44.01
RH Middle Frontal Gyrus	6	39	6	57	2.27	27.81
RH Middle Frontal Gyrus	8	30	24	48	2.22	29.7
RH Middle Frontal Gyrus	46	45	30	18	1.57	11.34
RH Middle Temporal Gyrus	22	69	−30	3	3.70	133.65
RH Middle Temporal Gyrus	21	66	−15	−3	3.70	150.39
RH Parahippocampal Gyrus	30	24	−51	6	3.10	20.52
RH Posterior Cingulate	31	9	−54	24	3.70	150.66
RH Posterior Cingulate	30	18	−54	18	3.22	26.19
RH Precentral Gyrus	6	48	−3	9	3.70	67.23
RH Precentral Gyrus	9	39	9	36	2.38	34.02
RH Superior Temporal Gyrus	22	60	3	−6	3.70	156.06
RH Superior Temporal Gyrus	13	51	−45	21	3.70	79.92
**(B) BETWEEN–GROUP RESULTS**						
LH Anterior Cingulate	24	0	36	6	1.47	8.37
LH Anterior Cingulate	32	−12	39	9	1.45	1.35
LH Caudate	Caudate head	−12	18	−9	3.70	109.35
LH Caudate	Caudate body	−15	12	18	3.70	28.62
LH Cingulate Gyrus	31	−6	−36	33	3.22	48.87
LH Claustrum	−	−27	24	9	3.70	74.52
LH Inferior Frontal Gyrus	11	−18	36	−18	3.70	60.21
LH Inferior Frontal Gyrus	46	−39	39	6	3.70	98.55
LH Inferior Frontal Gyrus	9	−42	9	21	3.70	66.15
LH Insula	13	−39	0	−6	3.70	262.44
LH Lentiform Nucleus	Putamen	−27	−15	3	3.70	18.63
LH Medial Frontal Gyrus	11	0	36	−12	3.70	76.95
LH Medial Frontal Gyrus	10	−3	57	6	3.70	29.16
LH Medial Frontal Gyrus	6	−3	−21	63	2.27	18.63
LH Middle Frontal Gyrus	9	−33	12	30	2.09	1.35
LH Paracentral Lobule	5	−3	−33	57	1.67	6.48
LH Parahippocampal Gyrus	30	−12	−36	6	3.70	26.19
LH Parahippocampal Gyrus	34	−15	−12	−15	3.10	7.56
LH Posterior Cingulate	29	−6	−45	18	3.10	7.83
LH Precuneus	31	−12	−51	27	1.54	1.35
LH Superior Frontal Gyrus	10	−12	69	−6	3.70	33.75
LH Superior Frontal Gyrus	11	−18	48	−15	3.70	38.34
LH Superior Temporal Gyrus	38	−48	12	−9	3.70	43.74
LH Transverse Temporal Gyrus	41	−33	−27	9	3.70	14.31
RH Anterior Cingulate	25	6	18	−3	3.70	39.96
RH Anterior Cingulate	24	3	24	18	1.33	1.08
RH Caudate	Caudate head	18	24	0	3.70	50.22
RH Inferior Frontal Gyrus	47	48	24	−3	3.70	71.01
RH Inferior Frontal Gyrus	13	45	21	6	3.70	26.19
RH Inferior Frontal Gyrus	46	51	45	0	2.09	2.97
RH Inferior Frontal Gyrus	9	51	12	24	1.52	3.51
RH Insula	13	45	6	0	3.70	76.14
RH Medial Frontal Gyrus	6	9	−18	51	3.70	233.28
RH Medial Frontal Gyrus	11	3	57	−18	2.09	2.43
RH Middle Frontal Gyrus	8	39	21	45	3.70	52.65
RH Middle Frontal Gyrus	11	30	39	−9	3.70	75.87
RH Precentral Gyrus	6	36	−9	63	3.22	34.29
RH Precentral Gyrus	4	36	−15	51	2.10	33.21
RH Superior Temporal Gyrus	22	45	−6	−3	3.70	27
RH Thalamus	−	3	−6	6	3.70	39.69

## Discussion

The present longitudinal study investigated gray matter volume changes over a 2 year period in emerging adult college students who were either sustained light-moderate drinkers or who had continued heavy alcohol use. Consistent with our initial hypotheses, we noted significant brain GMV loss in both groups. However, a direct two group comparison showed a significantly greater rate of loss in students with a sustained pattern of heavy alcohol use over 2 year follow-up. Moreover, this pattern of greater gray matter decline in heavy users occurred largely in regions responsible for emotion, memory, mental flexibility, and decision making, all of which might have a direct impact on student college success (Best et al., 2011).

It is important to recall that cortical pruning and restructuring reflects a normal, beneficial process during adolescence and young adulthood (Gogtay et al., 2004). Although the results noted in abstainers/light drinkers might closely reflect normal developmental processes, the exaggerated GMV-c due to continued heavy drinking would not be fully attributable to such cortical maturation. One possible interpretation could be that alcohol use leads to excess gray matter volume loss similar to that observed in adult alcoholics (Suzuki et al., 2010; Ersche et al., 2013; Sjoerds et al., 2013; Segobin et al., 2014; Droutman et al., 2015). The above hypothesis is supported by animal research that suggests alcohol interferes with neural stem cell proliferation leading to neurodegeneration and changes in brain structure and function (Morris et al., 2010). Animal reports have also shown marked differences in survival of cortical-cells in adolescent animals that were exposed to alcohol compared to controls (Crews et al., 2006; Hansson et al., 2010; Koss et al., 2012). Consistent with the above findings, changes observed in human macro-structural MRI studies such as ours may thus reflect tissue loss or remodeling related to inhibition of cell generation and survival. Preclinical studies also suggest that heavy alcohol intoxication might trigger microglia activation, oxidative stress, and pro-inflammatory changes which in turn leads to neurotoxic degeneration and/or prevents genesis of neurons and glia (Crews and Nixon, 2009; Alfonso-Loeches et al., 2012). Our results are also very consistent with a recent study that used region of interest based analysis to investigate similar longitudinal gray and white matter changes in adolescents before and after they initiated heavy alcohol use (Squeglia et al., 2015). That study observed a similar pattern of accelerated GMV-c in heavy alcohol initiators along with an attenuated growth of several white matter structures. The current study expands on these findings, demonstrating that continued heavy drinking in emerging adulthood leads to excessive gray matter loss in brain structures relevant to everyday functioning. Also, compared to the above study, we employed a voxel-wise whole brain analysis that should enable us to visualize GMV-c with greater spatial specificity.

Previous studies have shown that disruptions to adolescent brain maturation might adversely impact developmental cognitive and motor performance (Burgaleta et al., 2014). In the current study we noted both baseline decreases and accelerated GMV decline of the parahippocampal gyrus (a region primarily responsible for memory function) in the heavy drinking group, which is consistent with and expands results from cross-sectional reports showing reduced GMV in this medial temporal region for both individuals with alcohol dependence and those with family history of alcohol dependence (Suzuki et al., 2010; Sjoerds et al., 2013), The significant interaction of alcohol use on gray matter volume development as shown here might therefore help explain previous longitudinal studies showing worse attention, working memory, and visuospatial performance in adolescents after initiating alcohol use (Tapert et al., 2002; Hanson et al., 2011).

Interestingly, many of the significantly affected regions overlap with brain systems involved in reward, decision making, emotion and stress, that have been directly linked to addictive behavior (Schoenbaum and Shaham, 2008; Sinha, 2011; Droutman et al., 2015; O'Connor and Kenny, 2015; Silveri et al., 2016). Previous functional studies have shown increased activation to alcohol cues in medial frontal/ACC that underlie attention and motivation (Myrick et al., 2004; Dager et al., 2013). Altered interactions between ACC and parts of the striatum (also implicated in our current study) have shown to be related to motivational shift toward drug-induced cues, thus suggesting potential biological markers of addictive pathology (Myrick et al., 2004; Dager et al., 2013). Another key region identified in our study was inferior frontal gyrus (IFG), which functionally signals drug availability and triggers anticipation of use (Schoenbaum and Shaham, 2008). IFG over-reactivity has also been prospectively linked to escalating drinking in a previous study that used a subset of the current dataset (Worhunsky et al., 2016). It is interesting to note that our supplementary analyses show that some of these frontal differences existed at baseline and seemingly became worse over time. Insula, another region that showed exacerbated GMV loss in our heavy alcohol users, has been shown to play an important role in drug addiction (Droutman et al., 2015). Meta analyses of structural abnormalities in addicted individuals have described significantly reduced insular volume (Ersche et al., 2013). Functional studies have repeatedly observed blunted insular activity in substance-dependent individuals when engaged in decision-making tasks, and have also been shown to predict relapse (Sinha, 2011). The substantial overlap of excess gray matter loss with critical brain systems implicated in addiction might suggest that some of these differences may have been pre-existing. Compulsive drug taking due to poorly modulated decision making as a consequence of inability to learn from negative consequences is a hallmark of addiction. Baseline differences noted in our study were primarily limited to the pre-frontal cortex (PFC) which plays a key role in such decision making processes among other critical executive functions (George and Koob, 2010). Indeed, frontal lobe volumetric differences may be both a pre-existing risk factor as well as consequence of heavy alcohol use in adolescence (Silveri et al., 2016). It is also possible that such volumetric changes might contribute to differential neurochemical transmission sensitivity to alcohol that might in-turn enhance vulnerability to addictive-like behaviors into young adulthood. Given the longitudinal design here, our findings support the view that exacerbated gray matter loss is a consequence of continued heavy use. Although sustained heavy use might have an adverse impact on neurodevelopment, many studies in adolescent and adult alcoholics have demonstrated that certain brain structures and pathways show potential for recovery with alcohol abstinence or curtailment (Gazdzinski et al., 2005; Durazzo et al., 2011, 2016; Dresler et al., 2012; Segobin et al., 2014). Future longitudinal studies should determine the degree of volumetric recovery if drinking is reduced during earlier phases of brain development.

A number of additional factors in college including illicit substance use, stress levels, academic performance, peer pressure and psychiatric status could have an interactive effect with alcohol consumption (Meda et al., 2017). Although the concurrent use of other commonly abused substances such as marijuana were not analyzed here (due to excessive missing rate ~40%), prior studies that performed similar analyses to ours noted no significant interactions between alcohol and drug use with gray matter loss patterns (Squeglia et al., 2014). We measured personality and psychiatric metrics such as the STAI, BDI, MDD, and ADHD and found them to be balanced across our diagnostic groups. Also, cigarette smoking, which is often co-morbid with alcohol use and has been associated with gray matter changes (Franklin et al., 2014) was not significantly different between our groups. The proportions of smokers/non-smokers in our sample can be seen in Table 1. Given that previous cross-sectional studies have suggested differential alcohol effects exerted across sexes (Squeglia et al., 2014, 2015), we looked at potential group by sex and group by sex by time interactions in our dataset, but found none.

Despite its current strengths, our study had several limitations to consider. We did not have sufficient numbers of individuals who transitioned from heavy to light or light to heavy alcohol use, and were therefore unable to examine gray matter changes associated with other trajectories of alcohol use that are of great interest. We also did not capture longitudinal behavioral data that might help better understand relationships between GMV-c and cognitive functioning, which might be a good avenue for future studies. There may be other important interacting variables, such as stress levels, personality factors, or other substance use, which were not examined here but would be critical areas for future inquiries. We did not scan participants before the onset of drinking, making it difficult to determine whether gray matter volume differences were pre-existing; future studies such as the ongoing ABCD project will help delineate patterns of gray matter development in relation to varying patterns of substance use initiation and escalation.

## Conclusion

In summary, our study provides important longitudinal evidence regarding excessive gray matter loss in regions related to cognitive control, emotional regulation, and memory that might have both short and long-term implications on student life success. Importantly, regions of abnormal gray matter decline overlapped with those implicated previously in substance abuse, thus providing more evidence toward the theory that initiation and continued alcohol use during young adulthood might confer vulnerability for future, ongoing substance dependence.

## Author contributions

SM and AD contributed to design, analysis, and drafting of the manuscript. KH, HT, SR, RW, CA, CF, and GP contributed to the study funding, design, and drafting of the manuscript.

### Conflict of interest statement

The authors declare that the research was conducted in the absence of any commercial or financial relationships that could be construed as a potential conflict of interest.
